# The Relationship between Dispositional Mindfulness and Relative Accuracy of Judgments of Learning: The Moderating Role of Test Anxiety

**DOI:** 10.3390/jintelligence11070132

**Published:** 2023-07-04

**Authors:** Yue Yin, Baike Li, Xiao Hu, Xiaolin Guo, Chunliang Yang, Liang Luo

**Affiliations:** 1 Collaborative Innovation Center of Assessment for Basic Education Quality, Beijing Normal University, Beijing 100875, China; yiny19@mail.bnu.edu.cn; 2 Institute of Developmental Psychology, Faculty of Psychology, Beijing Normal University, Beijing 100875, China; 201931061067@mail.bnu.edu.cn; 3 Beijing Key Laboratory of Applied Experimental Psychology, National Demonstration Center for Experimental Psychology Education, Faculty of Psychology, Beijing Normal University, Beijing 100875, China; bnuhx2010@foxmail.com; 4 State Key Laboratory of Cognitive Neuroscience and Learning, Beijing Normal University, Beijing 100875, China

**Keywords:** judgments of learning, metamemory accuracy, dispositional mindfulness, test anxiety, moderating effect

## Abstract

Research has demonstrated that metacognition accuracy is far from perfect. The accuracy of judgments of learning (JOLs) is of critical importance in self-regulated learning. To explore what factors constrain JOL accuracy, the current study focused on mindfulness, which is intimately related to metacognition and anxiety. A total of 203 undergraduates (198 valid samples) were recruited to determine the relationships among five dimensions of dispositional mindfulness, test anxiety, and relative accuracy of JOLs. Results revealed that the interaction term for acting with awareness and test anxiety significantly predicted JOL accuracy. Further analyses indicated that for individuals with high test anxiety, but not for those with low test anxiety, acting with awareness positively predicted JOL accuracy. Considering that dispositional mindfulness is modifiable, these results help to inspire researchers to further explore whether mindfulness training can be used as a remedy to improve JOL accuracy.

## 1. Introduction

Judgments of learning (JOLs), a well-studied form of prospective metamemory monitoring, refer to the metacognitive estimates about the likelihood of successfully remembering a given item on a future memory test (Nelson and Narens 1990; Rhodes 2016; Yang et al. 2021). Typically, individuals regulate their learning activities (e.g., learning strategy selection, study time allocation) according to their JOLs (Metcalfe and Finn 2008; Yang et al. 2017). Therefore, the accuracy of JOLs is of critical importance for being a successful learner. However, previous studies have shown that JOL accuracy is far from perfect (Nelson and Dunlosky 1991; Yang et al. 2023). Inaccurate JOLs may lead to inadequate learning of course materials in the case of stopping learning prematurely, and may also lead to poor learning efficiency when individuals unnecessarily expend extra efforts toward processing well-mastered materials. Therefore, it is important to explore what factors constrain JOL accuracy.

Mindfulness, which is described as focusing one’s attention on current experience in a nonjudgmental or accepting way (Baer et al. 2006; Brown and Ryan 2003), is closely related to metacognition in terms of both neuropsychological findings and theoretical conceptualizations (for detailed discussion, see Jankowski and Holas 2014). Dispositional mindfulness is a state-like variable (Bishop et al. 2004) that can be developed with training (Schmidt et al. 2019; Sousa et al. 2021; Vorontsova-Wenger et al. 2022). Numerous studies have confirmed that mindfulness training is an effective intervention in many circumstances, such as promoting health and sustaining attention (Grossman et al. 2004; Khoury et al. 2015; Schumer et al. 2018; Verhaeghen 2020). Mindfulness training can also improve introspection of cognitive, affective, and experiential states (e.g., Baird et al. 2014; Lutz et al. 2007). However, few empirical studies have examined the relationship between the two psychological components—mindfulness and metacognitive monitoring (Baird et al. 2014; Schmidt et al. 2019).

To our knowledge, by far only two behavioral experiments have been conducted to explore the effect of mindfulness training on the accuracy of confidence ratings (i.e., retrospective judgments). Baird et al. (2014) recruited 50 undergraduates and randomly assigned them to either a mindfulness or a control group. Across two weeks, participants in both groups met for 45 min, four times per week. Those in the mindfulness group performed the focused attention meditation, whereas those in the control group attended nutrition courses. To measure the accuracy of confidence ratings, both groups were instructed to complete a memory task and a perceptual task before (pre-assessment) and after (post-assessment) the intervention phase. In the memory task, participants were asked to study 160 words one-by-one and then take a recognition test. During the recognition test, 320 words (160 old and 160 new words) were presented in random order and participants judged whether each word was *old* or *new*. Participants then reported their confidence regarding the correctness of their answers on a scale ranging from 1 (*low confidence*) to 6 (*high confidence*). The results showed that relative accuracy (i.e., meta-*d′/d′*) of confidence ratings in the memory task was significantly improved by mindfulness training (*p* < .05), whereas nutrition training had no statistically detectable influence (*p* = .24).

Schmidt et al. (2019) further explored whether and which kind of mindfulness trainings could improve the accuracy of confidence ratings. In their experiment, participants in the mental monitoring (MM) group were trained to focus attention on pure mental contents (i.e., internal cues). In contrast, those in the self-observation of the body (SoB) group were trained to focus attention on their own behavior (i.e., external-body cues). Across eight weeks, participants in both groups met once each week. The experimental procedure was similar to that of Baird et al. (2014), but the findings are substantially different from those observed by Baird et al. (2014). Specifically, Schmidt et al. (2019) observed that MM training had no statistically detectable influence on the relative accuracy of confidence ratings (*p* = .14). Intriguingly, SoB training significantly decreased the accuracy of confidence ratings (*p* < .05).

To sum up, previous studies explored the influence of mindfulness training on the accuracy of retrospective metamemory monitoring (i.e., confidence ratings). Baird et al. (2014) found that mindfulness training improved the relative accuracy of confidence ratings, whereas Schmidt et al. (2019) found that SoB training reduces the relative accuracy of confidence ratings. To our knowledge, by far, no research has been conducted to investigate the relationship between mindfulness and the accuracy of prospective metamemory monitoring (e.g., JOLs). Given that prospective and retrospective metamemory monitoring are distinct in nature (Nelson and Narens 1990) and prospective metamemory monitoring is of critical importance in self-regulated learning (Metcalfe and Finn 2008; Yang et al. 2017), it is necessary to explore the association between mindfulness and accuracy of prospective monitoring.

Anxiety, which is inversely related to mindfulness (e.g., Carsley and Heath 2020; Mesmer-Magnus et al. 2017; Prieto-Fidalgo et al. 2021; Shahidi et al. 2017), has a detrimental impact on cognitive performance (Eysenck et al. 2007; Putwain et al. 2015, 2013; Silaj et al. 2021). However, the relationship between anxiety and JOL accuracy has rarely been investigated. The current study focused on a specific type of trait anxiety closely related to learning and test performance, that is, test anxiety. Test anxiety is characterized as the tendency to appraise performance evaluative situations as threatening and react with excessive anxiety (Spielberger and Vagg 1995; Putwain and Symes 2020).

Several cognitive theories, including cognitive interference theory (Sarason 1988), processing efficiency theory (Eysenck and Calvo 1992), and attentional control theory (Eysenck et al. 2007), have been proposed to explain how (test) anxiety affects cognitive processes. Those theories suggest that, compared to individuals with low (test) anxiety, those with high (test) anxiety are more likely to generate negative thoughts (e.g., worry or self-preoccupation) unrelated to the current task, which may consume their limited attentional resources (Eysenck and Calvo 1992; Eysenck et al. 2007). Hence, when a cognitive task itself is highly demanding, individuals with high (test) anxiety will experience the feeling of “going blank” (Putwain 2009) and their task performance will be impaired.

As numerous studies have shown that mindfulness is conducive to sustaining individuals’ attention to what is occurring at the present (Baer et al. 2006; Bishop et al. 2004; Brown and Ryan 2003; Verhaeghen 2020), it is an opposite construct compared with anxiety to some extent (Arch and Craske 2010; Brown and Ryan 2003; Prieto-Fidalgo et al. 2021). That is, individuals with high test anxiety are usually accompanied by decreased attentional resources, whereas those with high dispositional mindfulness are good at focusing attention on the present moment (Bishop et al. 2004; Verhaeghen 2020). Hence, it is likely that high levels of dispositional mindfulness can remedy the undesirable consequences brought by high anxiety when individuals complete a demanding task. For instance, when participants are required to complete a memory task to measure JOL accuracy, high levels of dispositional mindfulness may counteract or compensate for the poor performance (i.e., low accuracy of JOLs) brought by high test anxiety. In other words, it is reasonable to speculate that for individuals with high test anxiety, dispositional mindfulness may positively correlate with JOL accuracy; for those with low test anxiety, however, dispositional mindfulness may play a minimal or even no role in the variability of JOL accuracy.

Indeed, previous studies have demonstrated that high levels of dispositional mindfulness serve as a protector (or a buffer) of anxiety (Jaiswal et al. 2019; Mankus et al. 2013), and the interaction between mindfulness and another independent variable (e.g., anxiety) can successfully predict clinical symptoms (e.g., Dolbier et al. 2021; Gorgol et al. 2022; Jaiswal et al. 2019; Mankus et al. 2013; Tubbs et al. 2019). For instance, Gorgol et al. (2022) found a significant interaction between mindfulness and eveningness in predicting anxiety symptoms, such that a higher level of dispositional mindfulness attenuated the relationship between eveningness and the presence of anxiety symptoms. Dolbier et al. (2021) found that as mindfulness increased, the relationship between adverse childhood experiences and generalized anxiety decreased. Additionally, Mankus et al. (2013) found that the relationship between mindfulness and heart rate variability was significantly moderated by generalized anxiety level. That is, only for individuals with high generalized anxiety, but not for those with low generalized anxiety, mindfulness was positively associated with heart rate variability. Based on the aforementioned studies, it is reasonable to assume that anxiety may moderate the relationship between dispositional mindfulness and JOL accuracy.

The current study aims to be the first to explore the relationship between dispositional mindfulness, (trait) test anxiety, and JOL accuracy. Exploring this question may shed new light on the relationship between mindfulness and metacognition, especially prospective metamemory monitoring. Meanwhile, elucidating the moderating role of test anxiety in the relationship between dispositional mindfulness and JOL accuracy is largely instructive for practical researchers, because they can treat mindfulness training as a potential approach to improve JOL accuracy for the targeted population. That is, if the positive correlation between mindfulness and accuracy of JOLs exists in individuals with high test anxiety but not in those with low test anxiety as we speculated above, then mindfulness training can be used to improve levels of dispositional mindfulness and JOL accuracy for the targeted population (Dolbier et al. 2021; Schmidt et al. 2019; Sousa et al. 2021; Vorontsova-Wenger et al. 2022).

## 2. Materials and Methods

### 2.1. Participants

A power analysis was conducted via G*Power (Faul et al. 2007). Assuming a small to medium effect size (*r* = 0.20), the power analysis estimated that 193 participants were sufficient to detect a significant (*α* = 0.05) correlation between dispositional mindfulness and the accuracy of JOLs at 0.80 power. Accordingly, 203 undergraduates were recruited from Beijing Normal University (BNU) and they received 60 RMB as compensation. Data from 5 participants were excluded due to constant JOL values or memory performance (see below), leaving final data from 198 participants (*M* age = 21.84 years, *SD* = 2.27; 73.20% female).

### 2.2. Materials

In total, 100 two-character Chinese words were selected from the Chinese word database developed by (Cai and Brysbaert 2010) to serve as learning materials used in the memory task. The word frequency ranged from 2.98 to 51.33 per million. Four words were used for practice and were excluded from data analyses. The remaining 96 words were used in the formal experiment and were randomly divided into 6 lists, with 16 words in each list. For each participant, the present sequence of words in each list and the list sequence were randomly decided by a computer. All stimuli were presented via the MATLAB *Psychtoolbox* package (Kleiner et al. 2007).

The Five Facet Mindfulness Questionnaire (FFMQ; Baer et al. 2006) is a 39-item questionnaire. FFMQ has been widely used to measure five dimensions of dispositional mindfulness, including *observing* (i.e., the tendency to notice internal and external stimuli, e.g., “*I notice how foods and drinks affect my thoughts, bodily sensations, and emotions*”), *describing* (i.e., expressing one’s internal experience in words, e.g., “*I’m good at finding the words to describe my feelings*”), *acting with awareness* (i.e., focusing on one’s current activities, and not behaving absentmindedly, e.g., “*I do jobs or tasks automatically, without being aware of what I’m doing*”), *non-judging of inner experience* (i.e., evaluating one’s experience in a non-judging way, e.g., “*I make judgments about whether my thoughts are good or bad*”), *non-reactivity to inner experience* (i.e., allowing internal experiences to come and go, without being absorbed, e.g., “*I perceive my feelings and emotions without having to react to them*”). The items are rated on a 5-point Likert scale ranging from 1 (*never or rarely true*) to 5 (*very often or always true*). In each subscale, a higher total score represents a higher level of mindfulness. The Chinese version of the FFMQ was used in this study, which has adequate internal consistency (Cronbach *α* = 0.75, 0.84, 0.79, 0.66, and 0.45 for *observing*, *describing*, *acting with awareness*, *non-judging,* and *non-reactivity*, respectively) and test–retest reliability (test–retest reliability = 0.74, 0.70, 0.44, 0.61, and 0.51 for *observing*, *describing*, *acting with awareness*, *non-judging,* and *non-reactivity*, respectively) (Deng et al. 2011).

The short form of the Test Anxiety Inventory (TAI) is a 5-item questionnaire measuring individual differences of trait anxiety in the academic context (e.g., “During examinations I get so nervous that I forget facts I really know”; Taylor and Deane 2002). The items are rated on a 4-point Likert scale ranging from 1 (*hardly never*) to 4 (*almost always*). A higher total score represents a higher level of test anxiety. The Chinese version of the short form of TAI was used in this study, with Cronbach *α* = 0.81 and test–retest reliability *r* = 0.81, *p* < .001 (Dong et al. 2011).

### 2.3. Procedure

The procedure consisted of two parts, including a memory task and the implementation of questionnaires. In the memory task, participants were informed that they would study six lists of words, and each list was followed by a free recall test. Before the formal experiment, participants were asked to complete a practice task (4 trials in total) to ensure that they fully understood the task requirement.

In the formal experiment, 96 words were randomly divided into 6 lists, with 16 words presented one at a time for 1500 ms in each list. After a given word was presented for study, a slider ranging from 0 (*Sure I will not remember it*) to 100 (*Sure I will remember it*) was presented on screen and participants were instructed to make a JOL to predict the likelihood of remembering the word on a later test by dragging and clicking the scale pointer. Immediately after studying each list, participants completed a distractor task in which they solved math problems (e.g., *7 + 8 = __?*) for 30 s. Participants were then given 90 s to write down the words they could remember on a blank page. After each free recall test, participants rested for 60 s and then moved on to the next list.

After completing all six lists of the memory task, participants were asked to complete the FFMQ and the short form of TAI. The presentation order of the two questionnaires was counterbalanced among participants.

### 2.4. Statistical Analyses

Goodman–Kruskal gamma is a prevailing indicator for measuring the relative accuracy of JOLs, which is obtained by comparing the number of concordant pairs (Nc) and that of discordant pairs (Nd) between JOL magnitude and memory performance. The calculation formula is (Nc-Nd)/(Nc + Nd) (Nelson 1984). When no variation in JOLs or memory performance can be detected, gamma is not computable because all of the pairs belong to neither the concordant nor the discordant pairs. Thus, for participants whose gamma is unavailable due to the lack of variability of data, their data had to be deleted from analyses (Roebers et al. 2021). In the current study, the mean of gammas for six blocks was used as the measurement of the relative accuracy of JOLs for each participant. For a given participant, if over 3 out of 6 lists were associated with not-computable gamma, his or her data were excluded from analyses. In total, 5 participants’ data were excluded, leaving final data from 198 participants.

Below we first report the results of bivariate correlations to preliminarily examine the associations among all variables. Then, a hierarchical regression analysis was conducted to examine the predictors (i.e., dispositional mindfulness, test anxiety, and their interaction terms) of the relative accuracy of JOLs when controlling for gender and age. Results of JOLs and memory performance are also reported below.

## 3. Results

### 3.1. Descriptive Statistics

Descriptive statistics and two-tailed bivariate correlations for variables measured in this experiment are presented in Table 1.

Both observing (*r* = −0.15, *p* = .030) and non-reactivity to inner experience (*r* = −0.18, *p* = .011) were negatively correlated with the relative accuracy of JOLs (i.e., gamma). The remaining three dimensions of dispositional mindfulness and test anxiety were not statistically correlated with gamma (*p*s > .05). Additionally, observing (*r* = 0.16, *p* = .021), describing (*r* = 0.15, *p* = .034), and non-reactivity to inner experience (*r* = 0.21, *p* = .003) were positively correlated with JOLs. The remaining two dimensions of dispositional mindfulness and test anxiety were not statistically correlated with JOLs. Observing was positively correlated with memory performance (*r* = 0.21, *p* = .003). No other statistically significant correlations were detected in memory performance.

It should be noted that the results of Pearson’s correlations reflected bivariate correlations between predictors and dependent measures without controlling for other confounding variables. In order to better uncover the predictors of the relative accuracy of JOLs, hierarchical regression analyses were performed.

### 3.2. Hierarchical Regression Analyses

#### 3.2.1. Relative Accuracy of JOLs

The results of hierarchical regression analysis (see Table 2) showed that gender and age explained 1% of the variance in gamma at step 1, *F*(2, 194) = 0.90, *p* = .409. At step 2, five dimensions of dispositional mindfulness contributed a significant 6% additional variance, *F*(5, 189) = 2.40, *p* = .039. At step 3, the additional variance explained by test anxiety was not statistically detectable, *F*(1, 188) = 0.10, *p* = .752. At step 4, the interactions term for five dimensions of dispositional mindfulness and test anxiety contributed a marginally significant 5% additional variance in gamma, *F*(5, 183) = 2.13, *p* = .064.

**Figure 1:**
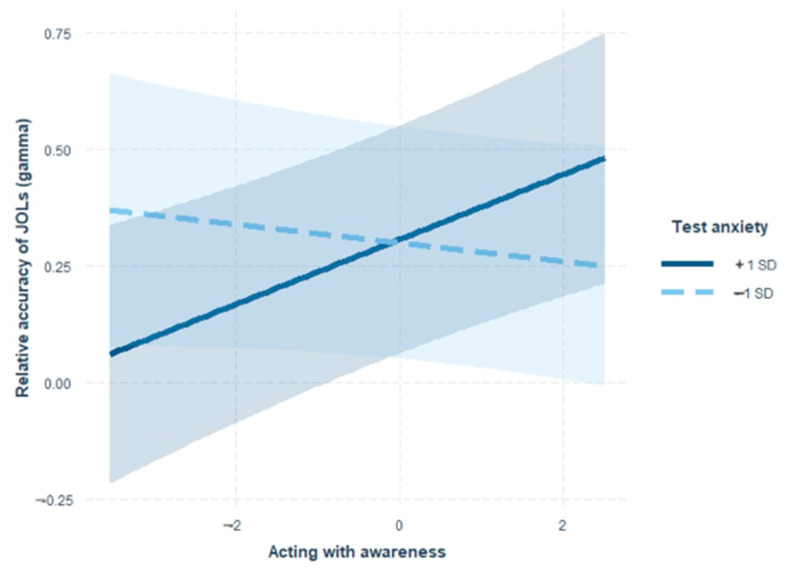
The moderating role of test anxiety in the association between dispositional mindfulness and relative accuracy of JOLs. The moderating effect is graphed for two levels of test anxiety: (1) high test anxiety (1 *SD* above the mean) and (2) low test anxiety (1 *SD* below the mean). Error bars represent 95% CI of the regression trend.

According to the model results of step 4, the interaction term for acting with awareness and test anxiety significantly predicted gamma when controlling for other variables (*b* = 0.05, *SE* = 0.01, *p* = .002). To explain the interaction effect, a simple slope test for the relationship between acting with awareness and gamma at low (1 *SD* below the mean) and high (1 *SD* above the mean) levels of test anxiety was conducted (see Figure 1). The results showed that for individuals with high test anxiety, higher levels of acting with awareness were linked to superior gamma (*b* = 0.07, *SE* = 0.02, *p* = .001). By contrast, for individuals with low test anxiety, there was no statistically detectable correlation between acting with awareness and gamma (*b* = −0.02, *SE* = 0.02, *p* = .297).

#### 3.2.2. JOLs

The results of hierarchical regression analysis (see Table 3) showed that gender and age explained 4% of the variance in JOLs at step 1, *F*(2, 194) = 4.11, *p* = .018. At step 2, five dimensions of dispositional mindfulness contributed a 5% additional variance, *F*(5, 189) = 2.09, *p* = .069. When test anxiety was included at step 3 and interaction terms were included at step 4, the additional variances explained by those variables were not statistically detectable (*p*s > .05). According to the results of step 4, five dimensions of dispositional mindfulness, test anxiety, and interaction terms did not significantly predict JOLs when controlling for gender and age.

#### 3.2.3. Memory Performance

The results of hierarchical regression analysis (see Table 4) showed that gender and age explained no variance in memory performance at step 1, *F*(2, 194) = 0.20, *p* = .816. At step 2, five dimensions of dispositional mindfulness contributed a significant 7% additional variance, *F*(5, 189) = 2.95, *p* = .014. When test anxiety was included at step 3 and interaction terms were included at step 4, the additional variances explained by those variables were not statistically detectable (*p*s > .05). According to the model results of step 4, observing positively predicted memory performance when controlling for other variables (*b* = 3.17, *SE* = 1.12, *p* = .005). The remaining dimensions of dispositional mindfulness, test anxiety, and interaction terms did not significantly predict memory performance (*p*s > .05).

## 4. Discussion

There is a scarcity of research examining the relationship between dispositional mindfulness and relative accuracy of JOLs, and potential moderators. The current study filled this important gap by showing that the interaction term for acting with awareness and test anxiety significantly predicted gamma (i.e., the relative accuracy of JOLs). Further analyses showed that for individuals with high test anxiety, acting with awareness was positively correlated with gamma; for those with low test anxiety, however, there was no statistically detectable correlation between these two variables. These findings are consistent with our a priori hypothesis.

Cognitive theories related to (test) anxiety (Eysenck and Calvo 1992; Eysenck et al. 2007; Sarason 1988) propose that individuals with high anxiety are more likely to generate negative thoughts unrelated to the current task than those with low anxiety, which may consume their limited attentional resources, then leading to worse task performance. Therefore, the accuracy of JOLs for individuals with high test anxiety may be negatively affected by reduced attentional resources. Given that mindfulness and anxiety are opposite constructs to some extent (Arch and Craske 2010; Brown and Ryan 2003; Prieto-Fidalgo et al. 2021), high levels of dispositional mindfulness may counteract or compensate for negative effects brought by high test anxiety, as reflected by the positive correlation between dispositional mindfulness and relative accuracy of JOLs in individuals with high test anxiety observed here. Similar to the result pattern observed here, Mankus et al. (2013) found that only for individuals with high generalized anxiety, but not for those with low generalized anxiety, mindfulness was positively associated with heart rate variability. These results help to inspire researchers to explore the boundary conditions under which mindfulness plays an essential protective role.

The current study found that acting with awareness was the only dispositional mindfulness facet that significantly interacted with test anxiety in predicting JOL accuracy, suggesting that it is the main driver of the protective effect. This finding emphasizes the importance of considering different facets of the complex construct of dispositional mindfulness to understand the relationships between mindfulness and other variables (Dolbier et al. 2021). Acting with awareness refers to the ability to focus on one’s current activities, not to behave absentmindedly. Consistently, the result of a meta-analysis conducted by Verhaeghen (2020) found that among the five dimensions of dispositional mindfulness, acting with awareness was the only facet that positively relates to attention performance (*r* = 0.07, Hedges’ *g* = 0.14 (0.02, 0.19)). Therefore, acting with awareness, rather than the remaining dimensions of dispositional mindfulness, plays a protective role in the impact of test anxiety on JOL accuracy.

One limitation is that although the interaction term for acting with awareness and test anxiety was significant in predicting the relative accuracy of JOLs, and post hoc power analysis (two-tailed, *α* = 0.05, *n* = 198, $f^{2}$= 0.06) showed that the power for detecting this effect was 0.91, additional variance explained by the five interactions was marginally significant (*p* = .064). Considering that the current study is the first to explore the relationship between dispositional mindfulness and JOL accuracy, additional work is required to further increase the sample size to test the stability of these results. In addition, the present study focuses on two trait factors that may affect JOL accuracy, namely dispositional mindfulness and trait anxiety, and neglects the role of state anxiety. Although many previous studies have used similar paradigms to examine the effect of trait anxiety on learning outcomes (Tse and Pu 2012; Yang et al. 2020), and there is a positive correlation between trait anxiety and state anxiety (Spielberger and Vagg 1995; Taylor and Deane 2002), exploring the interaction terms for mindfulness and state anxiety could provide additional insight into how mindfulness and anxiety jointly affect JOL accuracy in the current memory task.

Another limitation concerns the lack of intervention studies exploring whether mindfulness training would enhance JOL accuracy in individuals with high test anxiety. In educational settings, accurate monitoring of learning status is important for self-regulated learning, and intervening to improve the accuracy of monitoring has widespread benefits. However, previous results about whether (if so, how) metacognitive ability can be improved with training remain contradictory (Baird et al. 2014; Carpenter et al. 2019; Schmidt et al. 2019). Encouragingly, the current study suggests that for individuals with high test anxiety, but not for those with low test anxiety, acting with awareness positively predicted the relative accuracy of JOLs. Therefore, whether mindfulness training can be used as an intervention approach to improve the accuracy of JOLs should be further explored. Moreover, future studies should explore whether mindfulness training, especially the training to improve acting with awareness, can be used to achieve the goal of improving JOL accuracy in individuals with high test anxiety.

In addition to JOL accuracy results, the current study also reported relevant results for JOLs and memory performance. The results showed that dispositional mindfulness, test anxiety, and their interaction terms did not statistically predict JOLs, intriguingly, observing positively predicted memory performance when other variables were controlled. One of the goals of mindfulness training is to improve the ability of individuals to observe what is occurring, such as focusing on their breathing, perceptions, and thoughts (Anicha et al. 2012; Kabat-Zinn 2005). Observing is the ability to notice internal and external stimuli, which may contribute to completing memory tasks in the current study. Li et al. (2021) found that observing is positively correlated with working memory performance, and other studies have found similar results (Anicha et al. 2012; Parkinson et al. 2019). The evidence mentioned above may be instrumental in explaining the current results, that is, there is a positive correlation between observing and memory performance.

To conclude, the current study is the first to explore the relationship between dispositional mindfulness and JOL accuracy. More crucially, the observed findings reveal that for individuals with high test anxiety, acting with awareness positively predicts the relative accuracy of JOLs; for those with low test anxiety, there is no statistically detectable correlation between these two variables. Further research exploring whether mindfulness training enhances JOL accuracy in individuals with high test anxiety is needed.

## Figures and Tables

**Table 1:** Descriptive statistics and Pearson’s Correlations between variables.

	*M*	*SD*	1	2	3	4	5	6	7	8
1. Relative accuracy	0.23	0.16	—							
2. JOLs	57.82	10.40	−0.20 **	—						
3. Memory performance	55.16	13.25	−0.19 **	0.34 **	—					
4. Observing	24.89	5.16	−0.15 *	0.16 *	0.21 **	—				
5. Describing	24.56	5.21	−0.07	0.15 *	0.02	0.35 **	—			
6. Acting with awareness	27.12	5.13	0.12	0.02	−0.10	0.01	0.34 **	—		
7. Non-judging	24.01	4.63	0.10	−0.02	0.01	−0.17 *	0.19 **	0.45 **	—	
8. Non-reactivity	19.45	3.21	−0.18 *	0.21 **	0.12	0.32 **	0.19 **	−0.02	−0.11	—
9. Test anxiety	11.67	3.18	−0.05	−0.02	0	−0.01	−0.23 **	−0.41 **	−0.33 **	−0.06

Note: * *p* < 0.05; ** *p* < 0.01.

**Table 2:** Moderating effect of test anxiety on the relationship between dispositional mindfulness and relative accuracy of JOLs.

	Step 1			Step 2			Step 3			Step 4		
	*b*	*SE*	*β*	*b*	*SE*	*β*	*b*	*SE*	*β*	*b*	*SE*	*β*
Gender (female = 1)	0.03	0.03	0.08	0.02	0.03	0.06	0.02	0.03	0.06	0.01	0.03	0.02
Age	0	0.01	−0.03	0	0.01	−0.04	0	0.01	−0.04	0	0.01	−0.05
Observing				−0.02	0.01	−0.10	−0.02	0.01	−0.10	−0.02	0.01	−0.11
Describing				−0.01	0.01	−0.06	−0.01	0.01	−0.06	−0.02	0.01	−0.09
Acting with awareness				0.02	0.01	0.14	0.02	0.01	0.13	0.03	0.01	0.15
Non-judging				0	0.01	0.02	0	0.01	0.02	0.01	0.01	0.04
Non-reactivity				−0.02	0.01	−0.12	−0.02	0.01	−0.12	−0.02	0.01	−0.10
Test anxiety							0	0.01	−0.03	0	0.01	0.02
Observing × Test anxiety										0	0.01	0.03
Describing × Test anxiety										−0.02	0.01	−0.15
Acting with awareness × Test anxiety										0.05	0.01	0.32 **
Non-judging × Test anxiety										−0.02	0.01	−0.12
Non-reactivity × Test anxiety										0.01	0.01	0.09
Δ*R*^2^	0.01			0.06 *			0			0.05		

Note: * *p* < 0.05; ** *p* < 0.01.

**Table 3:** Moderating effect of test anxiety on the relationship between dispositional mindfulness and JOLs.

	Step 1			Step 2			Step 3			Step 4		
	*b*	*SE*	*β*	*b*	*SE*	*β*	*b*	*SE*	*β*	*b*	*SE*	*β*
Gender (female = 1)	−3.26	1.72	−0.14	−2.53	1.74	−0.11	−2.61	1.74	−0.11	−3.11	1.80	−0.13
Age	0.52	0.34	0.11	0.50	0.34	0.11	0.52	0.34	0.11	0.47	0.34	0.10
Observing				1.03	0.83	0.10	1.03	0.83	0.10	0.89	0.87	0.09
Describing				0.75	0.85	0.07	0.79	0.85	0.08	1.07	0.89	0.10
Acting with awareness				−0.07	0.85	−0.01	0.07	0.88	0.01	−0.09	0.90	−0.01
Non-judging				−0.20	0.85	−0.02	−0.13	0.86	−0.01	−0.20	0.88	−0.02
Non-reactivity				1.41	0.79	0.14	1.43	0.79	0.14	1.39	0.83	0.13
Test anxiety							0.46	0.82	0.04	0.57	0.86	0.06
Observing × Test anxiety										0.02	0.83	0
Describing × Test anxiety										0.83	0.84	0.09
Acting with awareness × Test anxiety										0.89	0.90	0.10
Non-judging × Test anxiety										−0.97	0.92	−0.10
Non-reactivity × Test anxiety										0.94	0.80	−0.10
Δ*R*^2^	0.04 *			0.05			0			0.02		

Note: * *p* < 0.05.

**Table 4:** Moderating effect of test anxiety on the relationship between dispositional mindfulness and memory performance.

	Step 1			Step 2			Step 3			Step 4		
	*b*	*SE*	*β*	*b*	*SE*	*β*	*b*	*SE*	*β*	*b*	*SE*	*β*
Gender (female = 1)	−0.56	2.23	−0.02	−0.67	2.23	−0.02	−0.60	2.24	−0.02	−1.64	2.31	−0.06
Age	−0.28	0.44	−0.05	−0.34	0.44	−0.06	−0.36	0.44	−0.06	−0.42	0.44	−0.07
Observing				3.08	1.07	0.23 **	3.08	1.07	0.23 **	3.17	1.12	0.24 **
Describing				−0.69	1.09	−0.05	−0.72	1.09	−0.05	−0.56	1.15	−0.04
Acting with awareness				−1.86	1.09	−0.14	−1.99	1.13	−0.15	−2.14	1.15	−0.16
Non-judging				1.93	1.09	0.15	1.86	1.11	0.14	1.71	1.12	0.13
Non-reactivity				0.92	1.01	0.07	0.90	1.01	0.07	0.55	1.06	0.04
Test anxiety							−0.44	1.06	−0.03	−0.44	1.10	−0.03
Observing × Test anxiety										−0.67	1.06	−0.05
Describing × Test anxiety										1.74	1.07	0.14
Acting with awareness × Test anxiety										1.09	1.16	0.09
Non-judging × Test anxiety										−1.50	1.18	−0.12
Non-reactivity × Test anxiety										−0.57	1.02	−0.05
Δ*R*^2^	0			0.07 *			0			0.03		

Note: * *p* < 0.05; ** *p* < 0.01.

## Data Availability

The data contained in this project are publicly available at Open Science Framework (https://osf.io/e5mnb/).
